# A Forward Genetic Screen Identifies Eukaryotic Translation Initiation Factor 3, Subunit H (eIF3h), as an Enhancer of Variegation in the Mouse

**DOI:** 10.1534/g3.112.004036

**Published:** 2012-11-01

**Authors:** Lucia Daxinger, Harald Oey, Anwyn Apedaile, Joanne Sutton, Alyson Ashe, Emma Whitelaw

**Affiliations:** Epigenetics Laboratory, Queensland Institute of Medical Research, Herston, Queensland, 4006, Australia

**Keywords:** mouse, epigenetics, forward genetic screen, eIF3h

## Abstract

We have used a forward genetic screen to identify genes required for transgene silencing in the mouse. Previously these genes were found using candidate-based sequencing, a slow and labor-intensive process. Recently, whole-exome deep sequencing has accelerated our ability to find the causative point mutations, resulting in the discovery of novel and sometimes unexpected genes. Here we report the identification of translation initiation factor 3, subunit H (*eIF3h*) in two *modifier of murine metastable epialleles* (*Mommes*) lines. Mice carrying mutations in this gene have not been reported previously, and a possible involvement of *eIF3h* in transcription or epigenetic regulation has not been considered.

Forward genetic screens have been used to identify genes involved in position effect variegation (PEV) in *Drosophila* (Eissenberg and Reuter 2009; Henikoff 1990; Schotta *et al.* 2003). PEV describes the variegated (red and white patches) eye phenotype caused by stochastic silencing of the *white* locus, when placed near pericentric heterochromatin (Muller 1930). Many of the genes involved in PEV turned out to have a critical role in gene silencing across the genome, and their functions are generally conserved between eukaryotes (Fodor *et al.* 2010). We have carried out a sensitized *N*-ethyl-*N*-nitrosourea (ENU) mutagenesis screen to identify genes involved in the establishment and maintenance of epigenetic state in the mouse. Our screen uses a GFP transgene that is expressed in red blood cells in a variegated manner due to stochastic transcriptional silencing. GFP expression is assessed by flow cytometry using one drop of blood. Approximately 55% of erythrocytes express the transgene in the wild-type transgenic line (Line3, an FVB/NJ-derived mouse strain). The percentage of cells expressing GFP and the mean fluorescence of expressing cells is highly reproducible among inbred Line3 individuals (Blewitt *et al.* 2005). Offspring of ENU-treated males are screened for a shift in GFP expression, and the screen is designed to detect both suppressors and enhancers of variegation (Blewitt *et al.* 2005). This is a screen for dominant effects, and mutants identified are called *modifiers of murine metastable epialleles* (*Mommes*). The screen has identified known (*Dnmt1*, *Smarca5*, *Hdac1*, *Baz1b*, *Trim28*) and novel (*Smchd1*) modifiers of epigenetic reprogramming, all of which play critical roles in normal embryonic, fetal, or early postnatal development (Ashe *et al.* 2008; Blewitt *et al.* 2008; Chong *et al.* 2007; Whitelaw *et al.* 2010). Here we report the identification of the mutated gene in *MommeD12* and *MommeD38*.

## Material and Methods

### Mouse strains and ENU screen

Procedures were approved by the Animal Ethics Committee of the Queensland Institute of Medical Research. The ENU screen was carried out in an FVB/NJ inbred line that carry a GFP transgene, as described previously (Blewitt *et al.* 2005). Mutant lines were maintained in this background, and all experiments were carried out using *MommeD* mice five generations or more removed from the *MommeD* founder.

### Whole-exome deep sequencing

The exomes of the two mutant lines, *MommeD12* and *MommeD38*, were captured using the SureselectXT Mouse All Exon version 1 kit (Agilent) and the SeqCap EZ Mouse Exome, version Beta 2 [110603_MM9_exome_rebal_2_EZ_HX1] kit (Roche NimbleGen), respectively. Both the DNA capture and library preparation was carried out as outlined in the protocols supplied by Agilent (protocol version 1.1.1) and Roche/nimblegen (NimbleGen SeqCap User’s Guide, version 1.0, Illumina optimized), except that a Bioruptor (Diagenode) was used for fragmentation of the DNA. The Bioruptor was optimized to produce ∼200–300 bp fragments, which was achieved by running for 3 × 10 min on the low setting with cycles of 30 s on and 30 s off. The resulting libraries were sequenced at a final concentration of 10 pM in a single lane each for 2 × 60 cycles on a GAIIx (Illumina) using the TruSeq PE Cluster Kit version 2 and TruSeq SBS kit version 5 GA (Illumina).

### SNP calling

For each of the two exomes, putative ENU mutations were identified by comparing variant calls from a mutant with variant calls from a control exome. The control exomes were prepared in parallel using the same capture kits and sequenced in the same deep-sequencing run. The variant calls were obtained by first mapping the deep-sequencing reads to the mouse reference genome (build 37, mm9) with the program BWA, version 0.6.1 (Li and Durbin 2009), using the default settings. The output was coordinate-sorted using SAMtools version 0.1.17 (Li *et al.* 2009), and PCR duplicates were eliminated using the program Picard MarkDuplicates version 1.48 (http://picard.sourceforge.net). The program SAMtools mpileup (Li *et al.* 2009), with the settings -q 20 -r chr15:40000000–68000000, was then used to generate pileup files from the linked region for the mutant and control exomes. Putative ENU mutations were finally identified with the program Varscan somatic version 2.2.8 (Koboldt *et al.* 2012) using the settings -min-coverage 15 and -min-var-freq 0.3. As input, the pileup files from a mutant and a wild-type were used, and mutations were identified as substitution or indel variants that differed between the two. Only a single heterozygous nucleotide substitution, subsequently validated using Sanger sequencing, was found for each of the mutant lines. These point mutations were validated in over 50 mutants and 50 wild-types in both cases.

### RNA isolation, cDNA analysis, and quantitative real-time RT-PCR

Total RNA was extracted from various tissues using TRI reagent (Invitrogen). cDNA was synthesized from total RNA using SuperScriptIII reverse transcriptase (Invitrogen). Quantitative real-time PCR was performed with the Platinum SYBR Green qPCR Super-Mix -UDG (Invitrogen) with primers designed to span exon/intron boundaries (available on request). All reactions were performed in triplicate and normalized to HPRT and GAPDH. PCRs were run on a Viia7 (Applied Biosystems).

## Results

**Figure 1:**
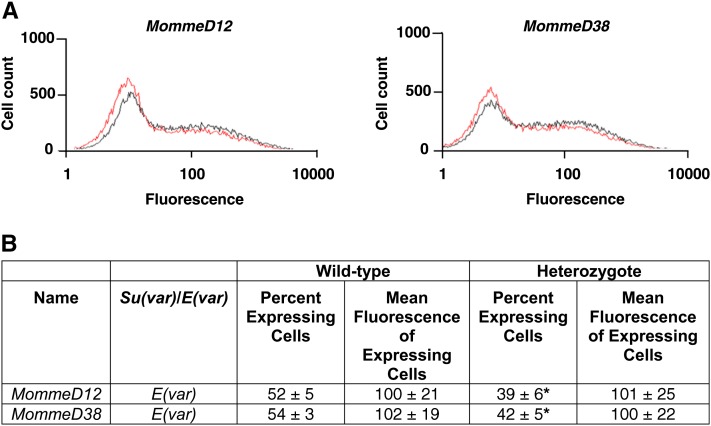
GFP expression profiles and mean fluorescence of expressing cells in *MommeD12* and *MommeD38*. (A) FACS profiles of *MommeD12* and *MommeD38* mutants. Erythrocytes from three-week-old mice were analyzed by flow cytometry with a GFP-positive gate set to exclude 99% of wild-type erythrocytes. In each case, the expression profiles from one litter are displayed. The phenotypically wild-type mice are shown in black and heterozygotes in red. The x-axis represents the erythrocyte fluorescence on a logarithmic scale, and the y-axis is the number of cells detected at each fluorescence level. Mean fluorescence was calculated using cells within the positive gate. Histograms depict only the GFP fluorescence channel. (B) Quantitative analysis of *MommeD12* and *MommeD38* expression and mean fluorescence of expressing cells. Each mutant line has a significantly different expression profile to that of wild-type littermates, reproducible over many generations. Data were collected from at least six litters in each case. Student *t*-test; *P ≤ 0.0001.

**Figure 2:**
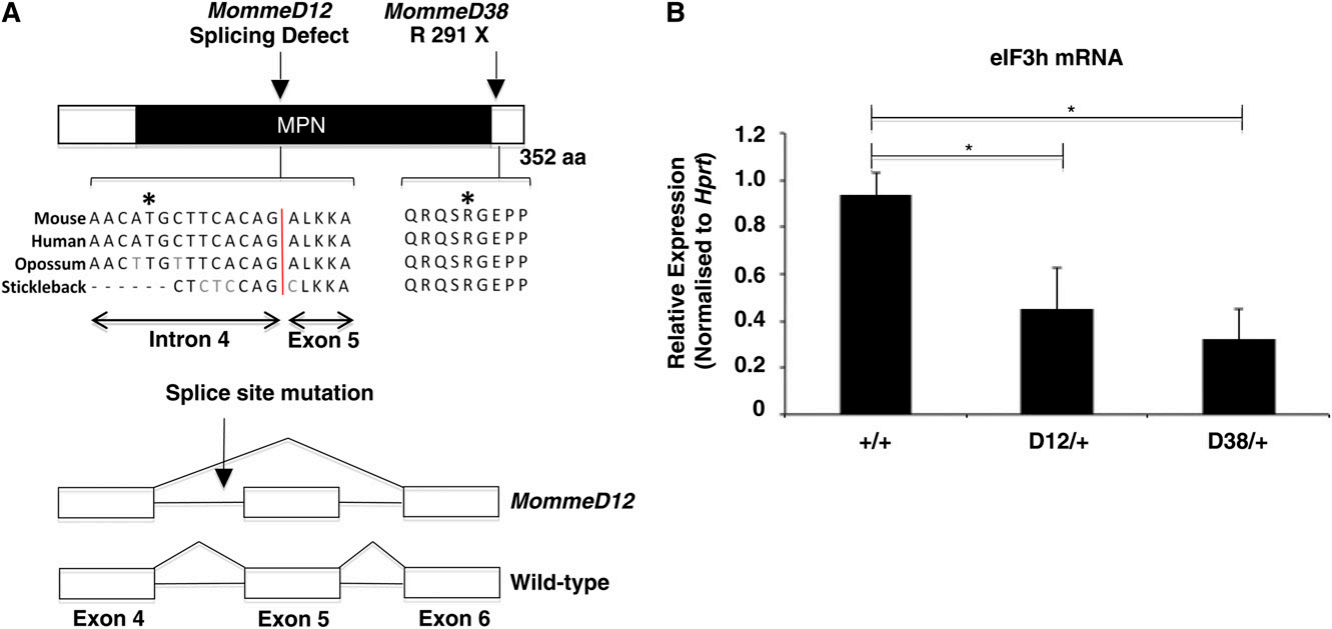
*MommeD12* and *MommeD38* have mutations in *eIF3h*. (A) Schematic of the eIF3h protein structure and positions of point mutations. The point mutation in *MommeD12* causes skipping of exon 5. The *MommeD38* mutation introduces a premature stop codon at amino acid 291, a highly conserved region of the protein. (B) Quantitative real-time RT-PCR analysis for *eIF3h* normalized to *Hprt*. The graph shows the mean ± SEM for four testes samples of each genotype. All reactions were performed in triplicate. Student *t*-test; *P < 0.05.

A shift in the percentage of cells expressing GFP was seen in *MommeD12* and *MommeD38* mutants. The percentage of cells was significantly lower than it was in wild-type mice, and the mutants were classified as enhancers of variegation (Figure 1). The level of mean fluorescence in the expressing cells did not differ between mutants and wild-types (Figure 1B). To identify the mutated genes in *MommeD12* and *MommeD38*, we generated a G2 mapping population by back-crossing *MommeD12* or *MommeD38* mice to *Line3C*, a C57BL/6J congenic mouse strain homozygous for the GFP transgene. Mapping by traditional microsatellite and SNP analysis identified a 3.3 Mb interval on chromosome 15 for *MommeD12* (Figure S1, A and C). Exome deep sequencing and bioinformatic interrogation of the linked interval, identified a T-to-A transversion in the conserved polypyrimidine tract 10 bp upstream of the 3′ splice site of intron 4 of *eIF3h*, a gene not previously considered to play a role in gene silencing (Figure 2A). Sequencing of the cDNA revealed that the mutation leads to skipping of exon 5 (Figure 2A), which results in an in-frame deletion of 50 highly conserved amino acids in the MPN (Mpr1-Pad1-N-terminal) domain.

An Illumina Golden Gate SNP genotyping assay was used to identify the linked chromosome for *MommeD38*. *MommeD38* mapped to an overlapping interval of 22 Mb (Figure S1, B and C). Subsequent exome deep sequencing and bioinformatic analysis of the interval identified a G-to-A transition in exon 7 of *eIF3h*. This mutation changes an arginine to a stop codon (Figure 2A).

For both *MommeD12* and *MommeD38*, putative ENU-induced substitution or indel variants within the linked intervals were identified by comparing exome variant calls generated from the two mutant lines with control exomes. The controls had been prepared in parallel using the same exome capture kits and on the same flow cell to minimize sample-to-sample variation. No other mutations were found in the intervals.

Both mutations lead to reduced *eIF3h* mRNA (Figure 2B), suggesting that *MommeD12* and *MommeD38* are null alleles. These are the first mutations reported in this gene in the mouse, and we designated these alleles *eIF3h^MommeD12^* and *eIF3h^MommeD38^*.

**Figure 3:**
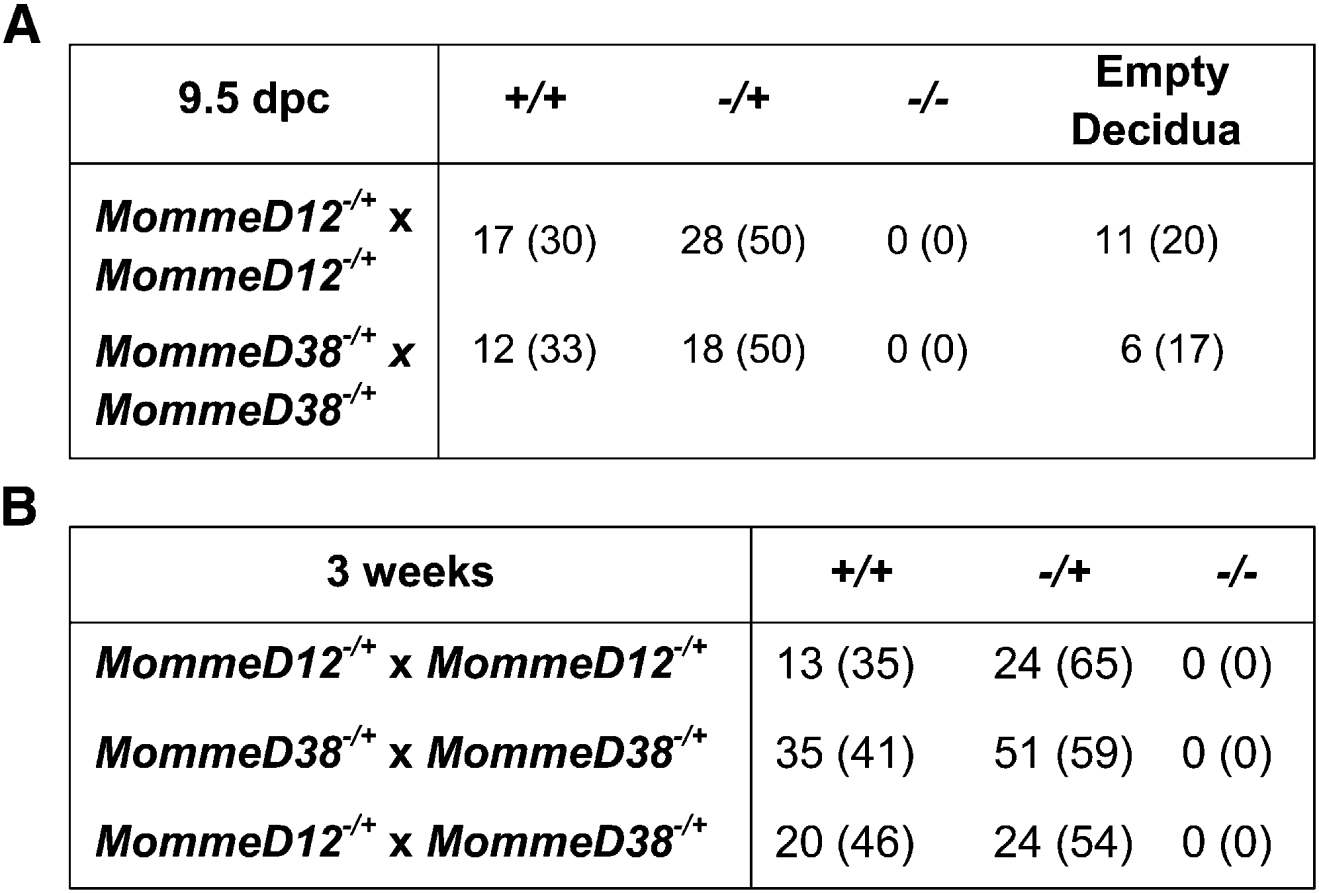
Embryo dissections and heterozygous intercrosses for *MommeD12* and *MommeD38*. (A) Embryonic dissections of *eIF3h* mutant mice. Embryonic dissections revealed no viable homozygotes at E9.5. All embryos were produced by natural matings, and detection of a vaginal plug was counted as embryonic day E0.5. The proportions of genotypes were compared with expected Mendelian ratios using a χ^2^ test. (B) Heterozygous intercrosses of *eIF3h* mutant mice analyzed at weaning. Tabulated data shows the number of observed mice and (percentage of total).

Heterozygotes for the *eIF3h^MommeD12^* and *eIF3h^MommeD38^* mutations were viable and fertile. However, for both *eIF3h^MommeD12^* and *eIF3h^MommeD38^*, heterozygous intercrosses produced no homozygous offspring at weaning, and timed matings revealed empty deciduas at E9.5 in ratios approximating that expected for the homozygous embryos (Figure 3A). Genotyping of the grossly normal embryos at E9.5 revealed that these were either wild-types or heterozygotes. Intercrosses between *eIF3h^MommeD12^* and *eIF3h^MommeD38^* heterozygotes produced no compound heterozygotes at weaning, as expected (Figure 3B). Our results indicate that eIF3h is required for normal embryonic development in the mouse. This is consistent with the finding that the eIF3h homolog in zebrafish is required for early embryonic development (Choudhuri *et al.* 2010).

## Discussion

eIF3h, together with two other nonconserved subunits (eIF3e and eIF3f) and three conserved subunits (eIF3a, eIF3b, and eIF3c), are thought to form the functional core of the mammalian eukaryotic translation initiation factor eIF3 (Masutani *et al.* 2007). Although it is possible that eIF3h has been identified because of an effect on the translation of the GFP mRNA, rather than transcription of the transgene, we consider it unlikely. We do not see a shift in the GFP mean fluorescence in the expressing cells in the *eIF3h^MommeD12^* and *eIF3h^MommeD38^* mutants, suggesting no major defect in GFP protein turnover (Figure 1B).

Some eIF3 subunits have been shown to interact with the 26S proteasome (Dunand-Sauthier *et al.* 2002; Hoareau Alves *et al.* 2002; Paz-Aviram *et al.* 2008; Yen and Chang 2000), suggesting that eIF3 has functions in biological processes other than translation initiation. Intriguingly, eIF3e, another nonconserved subunit of eIF3, has been shown to concentrate in the nucleus in mammalian cells in a cell cycle-dependent manner (Watkins and Norbury 2004), consistent with a role in the nucleus. This supports the hypothesis that eIF3h affects transgene expression via a role at the level of transcription.

In summary, we have identified the first two alleles of *eIF3h* in an unbiased forward genetic screen for modifiers of epigenetic reprogramming in the mouse. We anticipate that these mouse mutants will provide useful tools for others to perform further studies on eIF3h function in the cell. Interestingly, human eIF3h has recently been considered as a candidate gene for Microcephaly-Thin Corpus Callosum syndrome, a rare recessive disorder (Halevy *et al.* 2012).

## Supplementary Material

Supporting Information

## References

[bib1] Ashe A. Morgan D. K. Whitelaw N. C. Bruxner T. J. Vickaryous N. K., 2008 A genome-wide screen for modifiers of transgene variegation identifies genes with critical roles in development. Genome Biol. 9: R182 19099580 10.1186/gb-2008-9-12-r182 PMC2646286

[bib2] Blewitt M. E. Gendrel A. V. Pang Z. Sparrow D. B. Whitelaw N., 2008 SmcHD1, containing a structural-maintenance-of-chromosomes hinge domain, has a critical role in X inactivation. Nat. Genet. 40: 663–669 18425126 10.1038/ng.142

[bib3] Chong S. Vickaryous N. Ashe A. Zamudio N. Youngson N., 2007 Modifiers of epigenetic reprogramming show paternal effects in the mouse. Nat. Genet. 39: 614–622 17450140 10.1038/ng2031 PMC3199608

[bib4] Choudhuri A. Evans T. Maitra U., 2010 Non-core subunit eIF3h of translation initiation factor eIF3 regulates zebrafish embryonic development. Dev. Dyn. 239: 1632–1644 20503360 10.1002/dvdy.22289 PMC3089590

[bib5] Dunand-Sauthier I. Walker C. Wilkinson C. Gordon C. Crane R., 2002 Sum1, a component of the fission yeast eIF3 translation initiation complex, is rapidly relocalized during environmental stress and interacts with components of the 26S proteasome. Mol. Biol. Cell 13: 1626–1640 12006658 10.1091/mbc.01-06-0301 PMC111132

[bib6] Eissenberg J. C. Reuter G., 2009 Cellular mechanism for targeting heterochromatin formation in Drosophila. Int Rev Cell Mol Biol 273: 1–47 19215901 10.1016/S1937-6448(08)01801-7

[bib7] Fodor B. D. Shukeir N. Reuter G. Jenuwein T., 2010 Mammalian Su(var) genes in chromatin control. Annu. Rev. Cell Dev. Biol. 26: 471–501 19575672 10.1146/annurev.cellbio.042308.113225

[bib8] Halevy A. Basel-Vanagaite L. Shuper A. Helman S. Har-Zahav A., 2012 Microcephaly-thin corpus callosum syndrome maps to 8q23.2-q24.12. Pediatr. Neurol. 46: 363–368 22633631 10.1016/j.pediatrneurol.2012.03.014

[bib9] Henikoff S., 1990 Position-effect variegation after 60 years. Trends Genet. 6: 422–426 2087785 10.1016/0168-9525(90)90304-o

[bib10] Hoareau Alves K. Bochard V. Rety S. Jalinot P., 2002 Association of the mammalian proto-oncoprotein Int-6 with the three protein complexes eIF3, COP9 signalosome and 26S proteasome. FEBS Lett. 527: 15–21 12220626 10.1016/s0014-5793(02)03147-2

[bib11] Koboldt D. C. Larson D. E. Chen K. Ding L. Wilson R. K., 2012 Massively parallel sequencing approaches for characterization of structural variation. Methods Mol. Biol. 838: 369–384 22228022 10.1007/978-1-61779-507-7_18 PMC3679911

[bib12] Li H. Durbin R., 2009 Fast and accurate short read alignment with Burrows-Wheeler transform. Bioinformatics 25: 1754–1760 19451168 10.1093/bioinformatics/btp324 PMC2705234

[bib13] Li H. Handsaker B. Wysoker A. Fennell T. Ruan J., 2009 The Sequence Alignment/Map format and SAMtools. Bioinformatics 25: 2078–2079 19505943 10.1093/bioinformatics/btp352 PMC2723002

[bib14] Masutani M. Sonenberg N. Yokoyama S. Imataka H., 2007 Reconstitution reveals the functional core of mammalian eIF3. EMBO J. 26: 3373–3383 17581632 10.1038/sj.emboj.7601765 PMC1933396

[bib15] Muller H., 1930 Types of visible variations induced by X-rays in *Drosophila*. J. Genet. 22: 299–334

[bib16] Paz-Aviram T. Yahalom A. Chamovitz D. A., 2008 Arabidopsis eIF3e interacts with subunits of the ribosome, Cop9 signalosome and proteasome. Plant Signal. Behav. 3: 409–411 19704582 10.4161/psb.3.6.5434 PMC2634318

[bib17] Schotta G. Ebert A. Dorn R. Reuter G., 2003 Position-effect variegation and the genetic dissection of chromatin regulation in Drosophila. Semin. Cell Dev. Biol. 14: 67–75 12524009 10.1016/s1084-9521(02)00138-6

[bib18] Watkins S. J. Norbury C. J., 2004 Cell cycle-related variation in subcellular localization of eIF3e/INT6 in human fibroblasts. Cell Prolif. 37: 149–160 15030549 10.1111/j.1365-2184.2004.00305.x PMC6495725

[bib19] Whitelaw N. C. Chong S. Morgan D. K. Nestor C. Bruxner T. J., 2010 Reduced levels of two modifiers of epigenetic gene silencing, Dnmt3a and Trim28, cause increased phenotypic noise. Genome Biol. 11: R111 21092094 10.1186/gb-2010-11-11-r111 PMC3156950

[bib20] Yen H. C. Chang E. C., 2000 Yin6, a fission yeast Int6 homolog, complexes with Moe1 and plays a role in chromosome segregation. Proc. Natl. Acad. Sci. USA 97: 14370–14375 11121040 10.1073/pnas.97.26.14370 PMC18925

