# Supplementary material for: A Forward Genetic Screen Identifies Eukaryotic Translation Initiation Factor 3, Subunit H (eIF3h), as an Enhancer of Variegation in the Mouse
Source: G3 (Bethesda). 2012 Nov 1;2(11):1393–6. doi: 10.1534/g3.112.004036 (PMC3484669; doi:10.1534/g3.112.004036)
Supplement: Supporting Information [file supp_2_11_1393__index.html]

Supporting Information 

# A Forward Genetic Screen Identifies Eukaryotic Translation Initiation Factor 3, Subunit H (eIF3h), as an Enhancer of Variegation in the Mouse

## Supporting Information for Daxinger *et al.*, 2012

**Files in this Data Supplement:**

- Figure S1 - Linked genetic intervals for *MommeD12* and *MommeD38* (PDF, 141 KB)
