# Supplementary material for: A Forward Genetic Screen Identifies Eukaryotic Translation Initiation Factor 3, Subunit H (eIF3h), as an Enhancer of Variegation in the Mouse
Source: G3 (Bethesda). 2012 Nov 1;2(11):1393–6. doi: 10.1534/g3.112.004036 (PMC3484669; doi:10.1534/g3.112.004036)
Supplement: Supporting Information [file supp_2.11.1393_FigureS1.pdf]

A

Marker	Chr 15 location (bp)	Number of Mice				
		168	17	1	14	1
rs3251237	46091918					
rs31593385	51688047					
rs32382338	55001176					
D15Mit92	71050484					

B

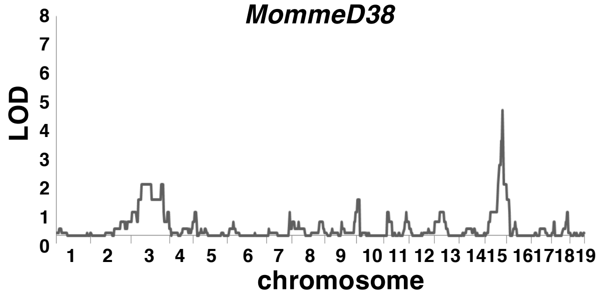

C

Name	Chromosome	Location	Linked interval (Mb)
MommeD12	15	51688047 - 55001176	3.3
MommeD38	15	45474639 - 67708702	22.2

Figure S1 Linked genetic intervals for *MommeD12* and *MommeD38*

(A) Linkage data for *MommeD12*. Table showing the SNP or Microsatellite marker, location on chromosome 15 (in base pair) and the number of mice used for mapping. The recombinants in this cross indicate *MommeD12* must lie between rs31593385 and rs32382338. Numbers indicate the number of recombinants at this position. DNA was isolated from G2 tails and a total of 201 tails was used for mapping.

(B) Manhattan plot showing the linked interval for *MommeD38*. The linked interval was identified by Illumina Golden Gate SNP genotyping analysis. Linkage analysis was performed on twelve wild-type and twelve heterozygous mice using the Mouse Medium Density Linkage panel. This contains 766 measurable SNPs between C57BL/6J and FVB/NJ. Samples were genotyped following the Illumina protocol. Genotype calls were made using the GenomeStudio v1.1 software. Only samples with a call rate >95 were accepted. The x-axis represents the chromosomes and the y-axis is the LOD score. A LOD score of >3 is significant.

(C) Table showing the chromosome, location on the chromosome and size of the linked intervals in Megabases (Mb) for *MommeD12* and *MommeD38*.
